# Enhanced faculty development: using the GMC survey to run a trainer development workshop

**DOI:** 10.1192/bjo.2021.435

**Published:** 2021-06-18

**Authors:** Martin Schmidt, Timothy Leung

**Affiliations:** 1 Surrey and Borders Partnership NHS Foundation Trust; 2 East London NHS Foundation Trust

## Abstract

**Aims:**

To investigate whether the General Medical Council (GMC) National Training Surveys (NTS) can be analysed to create a trainer development workshop that improves postgraduate training.

**Background:**

As part of its role in quality assurance of medical training, the GMC conducts an annual survey of trainers and trainees. The Trainer survey, part of the NTS, consists of 47 questions which are grouped into 11 indicators of quality. At Surrey and Borders Partnership NHS Foundation Trust, we were keen to use the comprehensive data in the NTS to improve training. We analysed each question to create a workshop to engage trainers in discussion about improving the experiences of trainers and trainees.

**Method:**

Our analysis of the NTS used data from the online reporting tool to calculate the scores that were obtained for each question in the 2018 NTS. A question was discussed at the workshop if it performed poorly relative to other questions in the indicator; to provide useful information; or to clarify ambiguity. Indicators where interesting comparisons can be drawn between the views of trainers and trainees were also discussed. The 90-minute workshop was led by the Leadership and Education Fellow and Director of Medical Education. Attendees were subsequently sent an online survey.

**Result:**

The workshop consisted of an introduction to the NTS; group discussion on which indicators were felt to be important, good- or poor-performing; discussion of specific questions; and a review of feedback from trainees.

12 questions and 3 indicators (Handover, Supportive environment, Rota design) were discussed. 11 questions were chosen for poor performance, which sought to contextualise the results within the experience of attendees. 8 questions were chosen to provide information, such as resources and current initiatives. 3 were chosen to clarify ambiguity. Many questions met several criteria.

17 attendees responded to the online survey. 64.7% agreed or strongly agreed that the NTS asks questions that are important for them. 76.5% agreed or strongly agreed that the NTS can be used to improve the trainer experience.

In the subsequent NTS, there was an improvement in 9/11 indicators in the Trainer Survey, with four green flags denoting performance in the top quartile of trusts nationally.

**Conclusion:**

The NTS can be used to structure a workshop that trainers feel can improve their experience. Our strategy demonstrates the value of analysing the NTS dataset intelligently to engage trainers in improving training.

